# Mesopredator Management: Effects of Red Fox Control on the Abundance, Diet and Use of Space by Feral Cats

**DOI:** 10.1371/journal.pone.0168460

**Published:** 2017-01-09

**Authors:** Robyn Molsher, Alan E. Newsome, Thomas M. Newsome, Christopher R. Dickman

**Affiliations:** 1 Department of Environment, Water and Natural Resources, Kangaroo Island, South Australia, Australia; 2 CSIRO, Lyneham, Canberra, Australian Capital Territory, Australia; 3 School of Life and Environmental Sciences, The University of Sydney, New South Wales, Australia; 4 Department of Forest Ecosystems and Society, Oregon State University, Corvallis, Oregon, United States of America; 5 School of Life and Environmental Sciences, Deakin University, Geelong, Victoria, Australia; 6 School of Environmental and Forest Sciences, The University of Washington, Seattle, Washington, United States of America; University of Alberta, CANADA

## Abstract

Apex predators are subject to lethal control in many parts of the world to minimize their impacts on human industries and livelihoods. Diverse communities of smaller predators—mesopredators—often remain after apex predator removal. Despite concern that these mesopredators may be 'released' in the absence of the apex predator and exert negative effects on each other and on co-occurring prey, these interactions have been little studied. Here, we investigate the potential effects of competition and intraguild predation between red foxes (*Vulpes vulpes*) and feral cats (*Felis catus*) in south-eastern Australia where the apex predator, the dingo (*Canis dingo*), has been extirpated by humans. We predicted that the larger fox would dominate the cat in encounters, and used a fox-removal experiment to assess whether foxes affect cat abundance, diet, home-range and habitat use. Our results provide little indication that intraguild predation occurred or that cats responded numerically to the fox removal, but suggest that the fox affects some aspects of cat resource use. In particular, where foxes were removed cats increased their consumption of invertebrates and carrion, decreased their home range size and foraged more in open habitats. Fox control takes place over large areas of Australia to protect threatened native species and agricultural interests. Our results suggest that fox control programmes could lead to changes in the way that cats interact with co-occurring prey, and that some prey may become more vulnerable to cat predation in open habitats after foxes have been removed. Moreover, with intensive and more sustained fox control it is possible that cats could respond numerically and alter their behaviour in different ways to those documented herein. Such outcomes need to be considered when estimating the indirect impacts of fox control. We conclude that novel approaches are urgently required to control invasive mesopredators at the same time, especially in areas where apex predators are absent.

## Introduction

Top-down processes can strongly influence the abundance, spatial distribution and behaviour of terrestrial predators via both interference competition and intraguild predation [1]. Interference competition occurs when individuals are directly antagonistic towards others, whereas intraguild predation is the killing and eating of species that use similar resources [2]. The effects of competition and predation may be symmetrical (both species compete against and kill each other) or asymmetrical (one species outcompetes and kills the other), but dominance is typically top-down and based on size [3,4]. The fitness of smaller predators, or mesopredators, should therefore be enhanced if larger, apex predators, are removed from the ecosystem, as predicted by the mesopredator release hypothesis [5].

An increasing body of evidence supports mesopredator release [6] and shows that its effects can be dramatic and affect a wide range of faunal elements [7]. However, most studies have focused on whether the removal of an apex predator results in the release of a single mesopredator [6]. While such research clarifies how apex predators regulate some ecosystems, diverse mesopredator communities often remain in other systems after the loss of an apex predator. In these situations, relationships between mesopredators are likely to shift [1], as are their top-down effects on prey [7], but these interactions have been little studied.

Mesopredators could fill the vacated ecological role of an apex predator. However, small and mid-sized mesopredators are often more numerous than apex predators and more diverse in their behaviour and resource use [8]. Competitive interactions between mesopredators may therefore differ from those between an apex predator and a mesopredator. Indeed, Donadio and Buskirk [9] suggested that killing between predators is more likely when the body size of the larger species is 2–5.4 times the mass of the victim. They also suggested that killing events between mammalian carnivores are more likely between species with high dietary overlap, and if the species have highly predatory habits [9]. An additional factor, however, is that mesopredators have been introduced into many ecosystems throughout the world [8]. Little is known about how these predators interact with each other, or with other components of the ecosystem [10], although the impacts of introduced predators on prey generally are much greater than those of their native counterparts [11].

In Australia, red foxes (*Vulpes vulpes*, ~6 kg) and feral cats (*Felis catus*, ~4 kg) were introduced following European settlement in 1788 and they both occur over the southern two-thirds of the continent. The dingo (*Canis dingo*, ~15 kg) was introduced some 3000–5000 years earlier [12]. There is evidence that dingoes can suppress or alter the behaviour of foxes [13], and possibly also cats [13,14]. However, widespread dingo eradication programmes, in conjunction with exclusion fencing, allow foxes and cats to live over large parts of Australia in the absence of the dingo [15]. Little is known about how foxes interact with cats, despite predation by these two species being major contributors to the ongoing wave of native mammal extinctions in Australia [16].

The potential for competition and intraguild predation between red foxes and feral cats is supported by studies that describe a high degree of dietary overlap between the two species [17–19]. Several studies suggest further that cats are probably subordinate to foxes. For example, Christensen and Burrows [20] observed a three-fold increase in cat abundance indices following dingo and fox control, while peaks in cat abundance indices have been recorded at times when fox abundance indices are low and prey availability is high [21,22]. Spatial segregation has also been found between the two predators, with mutually exclusive core areas despite overlapping home ranges [23]. Few studies have examined the dietary responses of cats to fox control. However, Risbey et al. [18] concluded that controlling foxes may lead to rises in cat numbers and consequent impacts on populations of small vertebrates at Heirisson Prong, Western Australia. A similar result was found at another site in Western Australia where cats became the dominant predator of woylies (*Bettongia penicillata*) at sites where foxes were controlled [24].

Here, we investigate the potential effects of competition from red foxes on feral cats by monitoring the abundance, diet, home range and habitat use of cats during a fox removal experiment at a study site where dingoes are absent. We also investigate potential intraguild predation by monitoring the diet of foxes over the same period. Evidence for competition would be adduced if, after fox control, cats (i) increase in abundance, or change their (ii) diet, (iii) home range size, or (iv) use of habitat. Although any change in parameters (ii)–(iv) after fox control could be taken as indicative of fox-cat interaction, evidence for competition would be strongest if cats shifted their use of space and other resources towards those used by foxes prior to their control. Evidence of intraguild predation would be adduced if cats occur in the diet of foxes. We use the results to discuss the management of foxes and cats on threatened fauna in situations where both predators co-occur in the absence of an apex predator.

## Materials and Methods

### Ethics statement

All sampling procedures and/or experimental manipulations, including lethal control of red foxes (baiting and shooting), was approved by ethics committees at The University of Sydney (L04/4-95/3/2116) and CSIRO (94/95-02). CSIRO and the Vertebrate Biocontrol CRC provided access to the Burrendong study region. This work did not involve lethal control or handling of endangered or protected species.

### Study area

**Fig 1:**
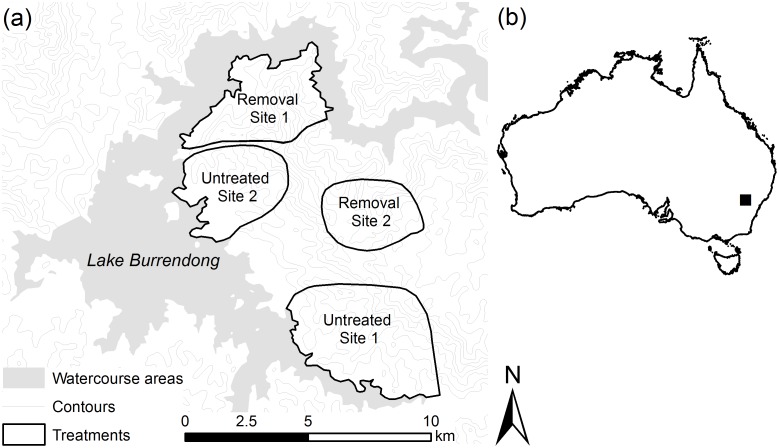
(a) Burrendong study site and treatments, and (b) location of the study site in Australia (black box). Red foxes were controlled (baited and shot) at removal sites 1 and 2 from October 1995 to August 1998, but left intact at untreated sites 1 and 2.

This study was conducted within a 50 km radius of the foreshores of Lake Burrendong (32°42S, 149°10E), in central New South Wales, Australia (Fig 1). The study area encompasses about 90 km^2^ of steep to undulating slopes that extend to the flat foreshore of the lake. The area has mild to cool average winter temperatures (2–15°C) and warm to hot summers (14–33°C) with annual average rainfall of 614 mm. During the study period, from July 1994 to August 1998, drought conditions prevailed and monthly rainfall was below average, although conditions improved in 1996. Feral cats and red foxes occur throughout the study area, and the dingo is absent. European rabbits (*Oryctolagus cuniculus*) were abundant until the arrival of Rabbit Calicivirus Disease (RCD) in June 1996. Common introduced species include the feral pig (*Sus scrofa*), house mouse (*Mus musculus*) and black rat (*Rattus rattus*). Common native species include the eastern grey kangaroo (*Macropus giganteus*), euro (*Osphranter robustus*), swamp wallaby (*Wallabia bicolor*), and the common brushtail possum (*Trichosurus vulpecula*). All of these species are potential prey for cats and foxes.

### Experimental design

Four sites (each 10–19 km^2^) were monitored within the study area (Fig 1). After 16 months, in October 1995, foxes were subject to lethal control at two of the sites, referred to as removal site 1 and 2 hereafter. The control program consisted of shooting all foxes observed on monthly visits using a low velocity 0.222-calibre rifle and burying 1080-poison baits at intervals of 200–400 m along ridge tops and unsealed roads [25]. At removal site 1 baits were set and replaced where necessary each month, but were exposed for 10 days every three months at removal site 2. Baiting continued at both sites until the end of the study. Although the two removal sites were not subjected to the same intensity of control, reductions in fox activity at both sites provided an effective experimental treatment [25]. Fox densities were not manipulated at the other two sites, referred to as untreated site 1 and 2 hereafter.

### Data collection

#### Abundance indices

The abundances of red foxes and feral cats were estimated at all four study sites using spotlight counts before (July 1994 to September 1995) and after fox control (October 1995 to August 1998). Counts were conducted using a 100 W spotlight from a vehicle travelling at < 10 km/h over three consecutive nights each month, although at untreated site 2 spotlight counts were conducted every three months for three consecutive nights. Moonlit, windy and rainy nights were avoided to reduce weather-related variability in counts. Transects followed the main access roads in each study site, with lengths varying from 5–7 km. Count data were expressed as mean numbers of target animals seen per kilometre for each three monthly interval.

#### Scat collections

Feral cat and red fox scats were collected on walks throughout the study sites in most months from July 1994 to June 1997. Scats were distinguished to species by size, shape, colour, texture and odour. Mammalian prey were identified from hairs using cross-sectioning and whole mount techniques [26]. If blowfly larvae were present in scats, the contents were classified as carrion. However, kangaroo, sheep and cattle remains were classified as carrion, irrespective of the presence of blowfly larvae because carcasses of these species were common in the study area and adults of these species are not common prey for foxes and cats [27,28]. Non-mammalian food items were identified using reference materials. For the cat scats, food items were sorted into five broad groups (rabbit, carrion, house mouse, possum, invertebrate) and percentage volume and percentage occurrence calculated. Percentage volume was defined as the proportion of the total volume of a scat that was occupied by a particular food item, while percentage occurrence was defined as the proportion of scats in a sample that contained a particular food item.

#### Feral cat home range size

Between November 1994 and August 1996, feral cats were trapped in all study sites in wire mesh cage traps and Victor soft catch leg-hold traps (Nos. 1, 1.5 and 3, Woodstream, Corp., Lititz, Pa., USA). Captured cats were weighed, sexed, and aged (young adult cat < 3 years, old adult cats ≥ 3 years) based on tooth wear and colour. Adult cats (females > 2.5 kg, males > 3.4 kg) were fitted with SIRTRACK or AVM 2-stage radio-transmitters attached to a neck collar. Radio-collared individuals were located with a handheld 3-element Yagi aerial and an AVM AR8000 receiver. At least 24 h were allowed between capture and initiation of radio-tracking to avoid possible bias caused by trapping and handling. Triangulation was used to estimate cat locations and involved taking two or more directional bearings. Consecutive locations were separated by ≥ 30 minutes to reduce autocorrelation. Each bearing was coded from 1–5, representing the confidence of the observer in the obtained bearing. Locations were then estimated from bearings using the program LOCATE II [29]. Bearings coded ≥ 3 were excluded if they distorted location estimates (determined visually), while bearings coded < 3 were not excluded regardless of their congruence with the other bearings.

#### Feral cat habitat use

Two methods were used to assess habitat use by feral cats. Firstly, macro and microhabitats were recorded at the locations where feral cat scats were collected. Macrohabitat categories included (i) callitris, (ii) grassland, and (iii) woodland. Microhabitats included (i) rabbit warrens, (ii) tracks, (iii) logs, (iv) carcasses, (v) bare ground, and (vi) den sites. Secondly, fixes from the home range analyses were assigned to four broad habitats based on their location. These habitats were delineated from aerial photographs and ground-truthing, and comprised (i) mudflats, (ii) grassland, (iii) open woodland, and (iv) open forest [23]. Fixes were overlaid with the digitized map to obtain the proportion of habitat types utilized by individual animals using RANGES V [30].

### Analyses

#### Abundance indices

Red fox abundance indices were initially converted to ratios, expressed as mean numbers of foxes observed per km over the period before fox control began in October 1995 divided by mean numbers observed per km for the period after fox control in each of the two removal sites. The same computations were carried out for the two untreated sites, with the mean numbers of foxes observed per km in the period before October 1995 divided by the mean numbers observed per km for the period after. Data for all four sites were log-transformed to equalize variances, and then subjected to a *t*-test (one-tailed) to check if continuous fox control had the expected effect of reducing fox activity in the removal sites. Although ratios can have unstable distributions, their use here allows for site-level replication (*n* = 2 removal sites and *n* = 2 untreated sites) and the use of a conservative but robust inferential test, without the need to use temporally non-independent runs of observations. Indices of feral cat abundance were analyzed in the same way.

#### Feral cat diet

The effects of red fox control on the percentage volume of the five prey groups in the diets of feral cats were examined using a generalized linear model, where treatment (removal sites *vs* untreated sites), fox control (before *vs* after) and study site were fitted to the model as factors. The effect of fox control on the occurrence of each prey group in the diets of cats was then tested using an analysis of deviance with binomial error and a logit link function using the same factors. For both analyses, each prey group was tested separately to avoid problems associated with the lack of independence between prey groups. For the percentage volume analysis, prey groups recorded infrequently in the diet were log-transformed (log X + 1) to reduce clumping of the residuals. Because multiple tests on the different prey categories increase the risk of committing type-1 errors, we interpret the dietary results with due caution.

#### Feral cat home range size

Home ranges of feral cats were calculated where ≥ 20 location fixes were obtained and an asymptote was reached with increasing numbers of fixes. Two methods were used initially to estimate home range size; however, only the 95% Minimum Convex Polygon (MCP) estimates were used because they were highly correlated with 95% kernel estimates (*r*^*2*^ = 0.78; *n* = 41; *P* < 0.001). The effects of red fox control on the home range sizes of cats were tested using generalized linear regression where the MCP estimate was the response variable, and study area, treatment, and fox control were fitted to the model as factors. In addition, season (winter 1995, summer 1995/96 and winter 1996), sex and age (young adult *vs* old adult) were fitted to the model as factors to examine their potential influences on home range size. To avoid possible confounding by seasonal effects, the analysis was repeated after excluding the summer 1995/96 period. In addition, a generalized linear regression (as above) was used to examine the effect of fox control on home range size of cats whose home ranges were estimated across all three seasons.

The effects of red fox control on day and night home range sizes of feral cats were also examined using generalized linear regression. Home range size was used as the response variable, and study site, treatment, time of day (day *vs* night), fox control, sex and age were fitted factors. Cats with < 10 fixes in either day or night ranges were excluded from the analysis. As above, analyses were conducted for cats in all three seasons, and then repeated with summer 1995/96 excluded.

#### Feral cat habitat use

The effect of red fox control on the percentage of feral cat scats in each macro- and microhabitat type was tested using analysis of deviance with a binomial error and a logit link function. The response variable was the percentage of cat scats in each habitat type, and treatment, fox control and study site were fitted to the model as factors. Each habitat type was tested separately.

The effect of red fox control on the overall habitat use of feral cats was examined using generalized linear regression where the response variable was the proportion of location fixes in each habitat type, and treatment and red fox control were fitted to the model as factors. Sex and age were also fitted to the model to examine possible interaction effects; summer habitat use was excluded to avoid possible confounding due to seasonal effects, but generalized linear regression was used to examine differences between day and night habitat use overall with all three seasons included in the analysis. The proportion of fixes in each habitat type was used as the response variable, and season, time of day, and study site were fitted to the model as factors.

### Intraguild predation

The occurrence of feral cat remains in red fox scats was used as a surrogate measure to determine whether red foxes routinely killed and ate cats during the study. We also provide the results from a concurrent study where fox stomach contents were assessed during the study period [28].

## Results

### Abundance indices

**Fig 2:**
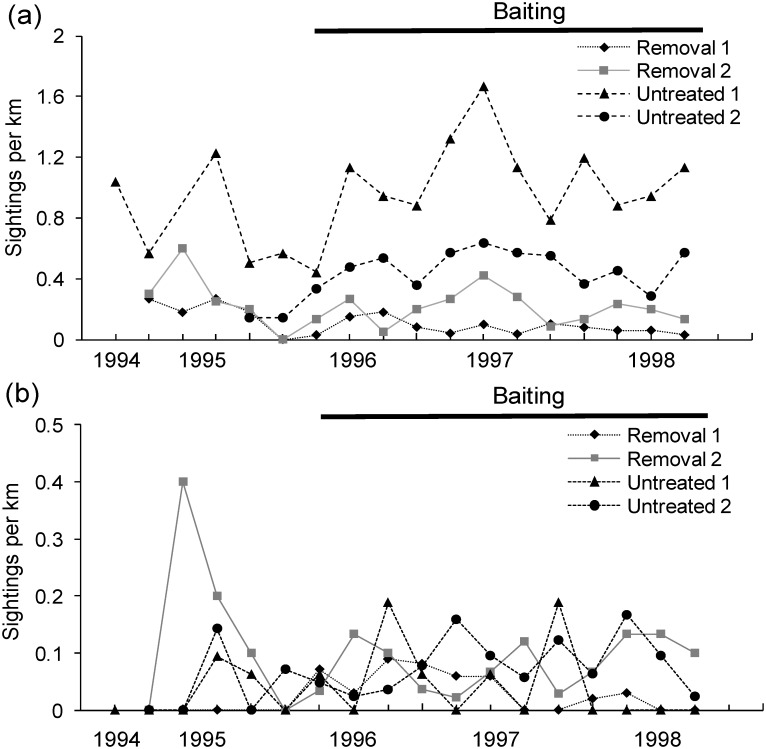
Abundance indices for (a) red foxes and (b) feral cats from July 1994 to August 1998 at Burrendong. Red foxes were baited and shot at removal sites 1 and 2 from October 1995 onwards, but left intact at untreated sites 1 and 2.

Red fox abundance indices were consistently lower at the removal sites relative to the untreated sites in the period after fox-removal began (*t* = 3.105, d.f. = 2, *P* = 0.045, 1-tailed). Fox numbers declined after fox control in October 1995 at removal site 1 and remained stable but low at removal site 2, while at the untreated sites fox numbers increased. Following fox control, fox abundance in the two removal sites was 3.8–10.6-fold lower on average than in the two untreated sites over the next 10 seasons (Fig 2). No change in feral cat abundance was detected after red fox control at the removal sites relative to the untreated sites (*t* = 0.028, d.f. = 2, *P* = 0.490, 1-tailed). Cat numbers were low at all sites and relatively stable throughout the study (Fig 2).

### Feral cat diet

**Fig 3:**
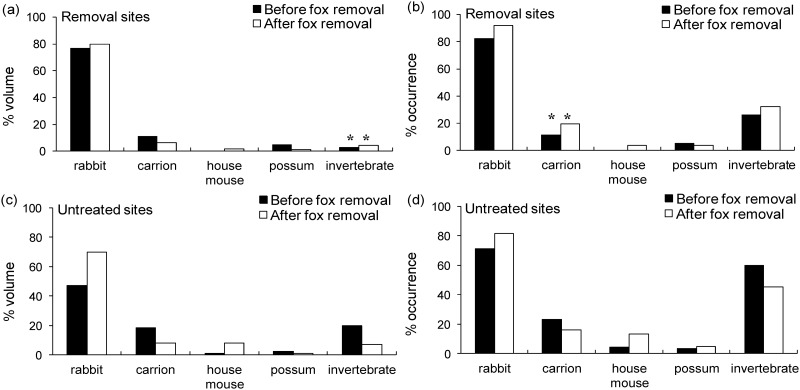
The effects of red fox control (before/after) on the importance of five major prey groups in the diet of feral cats (n = 408 scats) in removal sites ((a)/(b); *n* = 2) and untreated sites ((c)/(d); *n* = 2) at Burrendong. Data are pooled within removal and untreated sites. ** = a significant difference between removal sites and untreated sites (*P* < 0.05).

The volume of invertebrates increased in feral cat diet at the removal sites relative to the untreated sites (*F* = 4.28, d.f. = 1, 33, *P* = 0.046) (Fig 3), irrespective of seasonal effects (*F* = 1.94, d.f. = 3, 24, *P* = 0.149). The occurrence of carrion also increased in cat diet at the removal sites relative to the untreated sites (*F* = 21.39, d.f. = 1, 3, *P* = 0.02) (Fig 3). No differences were found for any of the other prey types tested (*P >* 0.05 for all tests).

### Feral cat home range size

**Fig 4:**
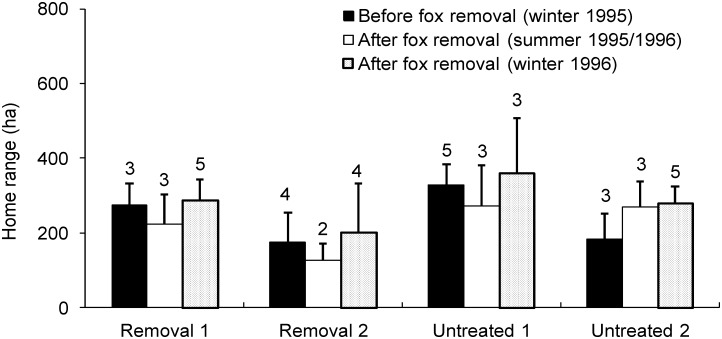
Red fox control effects on the mean home range size (95% Minimum Convex Polygon) for feral cats over three periods (+ standard errors) in sites where foxes were removed (removal sites, *n* = 2) and left intact (untreated sites, *n* = 2) at Burrendong. Sample sizes are shown above the bars.

There were no differences in the home range sizes of feral cats after red fox control between removal and untreated sites when all three seasons were included in the analysis (*F* = 0.01, d.f. = 1, 39, *P* = 0.908) (Fig 4). However, weak interaction effects were found with age (*F* = 3.85, d.f. = 1, 31, *P* = 0.059), but not with sex (*F* = 1.64, d.f. = 1, 31, *P* = 0.210). The interaction with age was largely driven by old cats (> 3 years) increasing their home range sizes at the removal sites relative to the untreated sites, where they did not change. Home range sizes varied between the four sites but did not differ overall (*F* = 1.77, d.f. = 1, 39, *P* = 0.170).

When the summer 1995/96 was excluded from the analysis, no red fox control effects were detected on feral cat home range size (*F* = 0.00, d.f. = 1, 28, *P* = 0.965). However, when only those cats (*n* = 7) whose ranges were estimated for all three seasons were included in the analysis, home range size decreased at the removal sites after fox control, relative to the untreated sites where range sizes increased (*F* = 6.87, d.f. = 1, 12, *P* = 0.022). This response was less pronounced when summer 1995/96 was excluded from the analysis (*F* = 4.25, d.f. = 1, 5, *P* = 0.094).

When day and night ranges were analyzed separately, no red fox control effects on feral cat home range size were found (*F* = 1.24, d.f. = 1, 64, *P* = 0.270). In addition, no interactions with sex (*F* = 0.05; d.f. = 1, 56, *P* = 0.832) or age (*F* = 0.53, d.f. = 1, 48, *P* = 0.470) were detected. Similar results were found when summer was excluded from the analysis (*F* = 0.74; d.f. = 1, 40; *P* = 0.396), with no interactions with sex (*F* = 0.02, d.f. = 1, 40, *P* = 0.894) or age (*F* = 0.41, d.f. = 1, 32, *P* = 0.526).

### Feral cat habitat use

**Fig 5:**
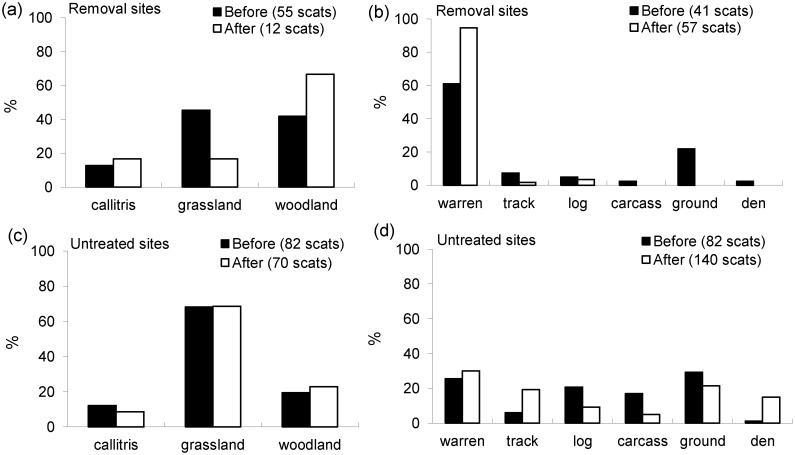
Red fox control effects (before/after) on the (a)/(c) macrohabitats and (b)/(d) microhabitats where feral cats deposited scats at Burrendong. Data are pooled within removal sites (*n* = 2) and untreated sites (*n* = 2).

Similar percentages of feral cat scats were found in the macro- and microhabitat types across all sites (*P >* 0.05 for all tests). However, fewer scats tended to be found in grassland habitats and more in woodland at the removal sites after red fox control (Fig 5). Similarly, more cat scats tended to be found at rabbit warrens at the removal sites after fox control, and fewer elsewhere. At the untreated sites, similar percentages of cat scats were found at rabbit warrens after fox control, but more scats were found on tracks, and fewer at hollow log entrances and carcasses compared to before fox control (Fig 5).

**Fig 6:**
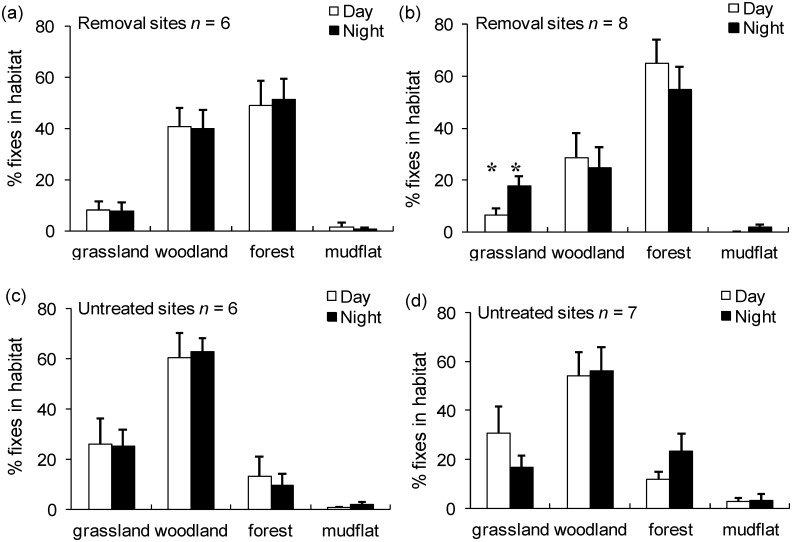
Differences in day and night feral cat habitat use before (winter 1995; (a)/(c)) and after (winter 1996; (b)/(d)) red fox control at Burrendong. Means with standard errors are shown. *n* = the number of cats tracked. ** = a significant difference between removal sites and untreated sites (*P* < 0.05).

After red fox control, no change in feral cat habitat use was detected overall at the removal sites relative to the untreated sites for any of the habitat types based on the location of fixes (*P* > 0.05 for all tests). No significant age or sex interactions were detected (*P* > 0.05 for all tests). When day and night periods were examined separately, there were no temporal differences in habitat use before fox control (winter 1995) at both the removal and untreated sites for any of the habitat types (*P >* 0.05 for all tests). However, after fox control (winter 1996), cats increased their use of grassland habitats at night at the removal sites, with the opposite trend at the untreated sites (*F* = 4.23; d.f. = 1, 26, *P* = 0.05). Similarly, open forest habitat tended to be used more often during the day at the removal sites, with the opposite trend at the untreated sites, but this difference was not significant (Fig 6).

### Evidence of intraguild predation

No feral cat remains were found in 343 red fox scats or in 255 red fox stomachs collected over the study period.

## Discussion

The results provide no indication that intraguild predation occurred between the two species of mesopredator, but suggest that the red fox affects some aspects of resource use by feral cats. Thus, while cats did not respond numerically to fox control, they did show modest shifts in their diet (Fig 3), range size and habitat use (Figs 5 & 6). These shifts occurred despite relatively weak fox removal, suggesting (1) that small changes in fox abundance can alter cat behaviour and (2) that cats may respond numerically and more significantly alter their behaviour under more intensive and sustained fox control regimes. We discuss the nature of the interaction between these two species in more detail below, and also the consequences generally for the broadscale management of competing mesopredators and the impacts they have on prey species.

Killing among mammalian carnivores is common and can account for up to 68% of known mortalities [4]. However, the frequency of attacks depends on dietary overlap and differences in body size [9]. In our study, red fox and feral cat diets overlapped by 73% in spring and summer and by 78% in autumn [23]. Such high overlap should have facilitated intraguild predation events, although we found no such evidence based on examination of fox scats and stomach contents. Subtle differences in prey use could have decreased killing events. For example, rabbits were more important in the diet of cats than foxes, particularly in summer when invertebrates and vegetation were relatively more prominent in the diet of foxes [23]. Differences in the diets of foxes and cats have been reported elsewhere. For example, Catling [17] found that rabbits comprised around 30% more of the weight of stomach contents of cats than of foxes. Catling [17] also suggested that both predators co-exist primarily by utilizing different-aged prey, with foxes mainly eating adult rabbits and cats young rabbits.

The mean (± SE) body weights we recorded for adult female and male feral cats were 3.34 ± 0.06 kg and 4.37 ± 0.14 kg, respectively [31], and those for adult female and male red foxes in the surrounding region were 4.52 ± 0.03 kg and 5.83 ± 0.05 kg, respectively (Saunders and McIlroy, unpublished data). On average, foxes were therefore less than twice the mass of cats in the Burrendong region. Thus, in addition to the subtle differences in diet noted above, the fact that foxes were not much larger than cats could also explain the lack of evidence we found for intraguild predation. A further possibility is that foxes killed cats, but did not eat them. In support of this, 17 radio-collared cats died during the study period, and where a cause of death could be determined from carcass examination (*n* = 8), three were attributed to fox attack based on teeth marks on radio-collars and puncture wounds detected on some cat bones [23]. Thus, while the number of killing events between foxes and cats is likely to have been low, killings cannot be ruled out completely, especially if our examination of scat and stomach contents underestimated the actual number of killing events.

Even if intraguild predation events are not common, competition between red foxes and feral cats could still play a role in regulating the abundance of these predators. However, we found no evidence for this despite fox abundance indices being lower at the removal sites relative to the untreated sites, and despite fox numbers increasing at the untreated sites (Fig 2). It is possible that the level of fox control in our study was not sufficient to allow cats to show a numerical response. For instance, complete or near elimination of foxes may have been necessary for cats to respond numerically. This occurred, for example, after the near elimination of foxes in Western Australia on the Peron Peninsula where cat numbers increased from approximately 300 to 950 following intensive fox control [32]. That result was partially attributed to an increase in cat births [32]; however, at Burrendong, all cat litters were born between September and March and no breeding was observed at other times [23]. Cat litter sizes also tended to decrease over time, although only two such observations were made [23]. This suggests that cat breeding was not altered by fox control at Burrendong. Additionally, rabbits were abundant at Burrendong until the arrival of RCD in June 1996. Rabbits can be staple prey for cats (> 80% occurrence in 499 scats collected at Burrendong), and thus the decline in rabbits after fox control (October 1995) could have prevented any numerical response by cats [27]. In other studies, peaks in cat numbers have been recorded at times when both fox numbers are low and prey availability is high [21,22], a combination of circumstances that did not occur at Burrendong. However, we acknowledge that the precision of our density estimates could have been improved if distance sampling methodology had been used [33], although low detection rates may have prohibited the calculation of fox and cat abundances, and detection functions are not always necessary when making comparisons between structurally similar landscapes [34].

Although feral cats did not respond numerically, we did uncover subtle changes to cat diets, home-ranges and use of habitat following fox control. These changes reflect weak levels of interference competition [2], and are potentially important because they could change the way that cats interact with their prey. In particular, cats showed relative increases in consumption of invertebrates and carrion after fox control at the removal sites relative to the untreated sites (Fig 3). The invertebrates eaten by cats were largely beetles (Coleoptera), cockroaches (Blattodea), and grasshoppers (Orthoptera) [27]. Based on volume, invertebrates were a minor component (< 10%) of cat diets, and considered supplementary prey. Cats may therefore have increased consumption of invertebrates simply because of differences in rates of consumption of other prey (Fig 3). However, because we found no differences in the occurrence or volume of other prey types consumed by cats between treatments, it is more likely that the increase in invertebrate consumption is linked to fox control, but the exact reasons for this are not clear.

A concurrent analysis of red fox diets in the surrounding area identified carrion as the most important food group for foxes by both volume (38.2%) and occurrence (73.8%) [28]. If fewer foxes were utilizing carrion, this resource may have been more readily available and accessible to feral cats, and thus explain why carrion occurred more frequently in cat diets after fox control at the removal sites (Fig 3). Because cats increased their use of carrion, a resource previously favoured by foxes, we adduce this as evidence that foxes can affect cat-resource use, although further investigation is required to understand the broader implications. For instance, if more carcasses were available to cats at fox-removal sites we might expect cat home-ranges to be smaller at fox-removal sites relative to untreated sites [35–37]. This was apparent in our study, but only when those cats (*n* = 7) whose home ranges were estimated for all three seasons were analyzed. We also found that the home range sizes of old cats (> 3 years) tended to increase after fox control at the removal sites, suggesting that cats in different age groups may respond differently to fox removal. However, it is possible that the exact volume and occurrence of carrion in fox and cat diets was not fully quantified because kangaroo, sheep and cattle remains were assumed to be carrion irrespective of the presence of blowfly larvae. Foxes are known to be significant predators of juvenile kangaroos and sheep [28], and the possibility of cat predation on these species cannot be ruled out. Consequently, the importance of carrion in fox and cat diet may have been overestimated, as the prevalence of predation on juvenile mammals was not known.

In terms of habitat shifts, we found that feral cats increased their use of grassland habitats at night after red fox control at the removal sites, and that open forest habitat tended to be used more often during the day at the removal sites (Fig 6). No other study has documented similar shifts in habitat use by cats, but habitat heterogeneity is increasingly being recognised as an important factor regulating competition between predators. For instance, species with low competitive ability can persist in a heterogeneous environment by making use of competition refuges [38]. Such refuges include areas with lower competitor densities, but could equally include habitats that do not favour contests for resources. Open habitats potentially favour contest competition because prey, including carcasses, can be located easily [39]. As such, it is perhaps unsurprising that cats in our study increased their use of open habitats and carcasses following fox control in the removal sites. Although we found fewer cat scats in grassland habitats at the removal sites following fox control (Fig 5), scat locations probably do not provide an accurate measure of habitat use, especially as scats are deposited for other reasons such as to mark territories.

In conclusion, our results suggest that red foxes can affect some aspects of feral cat behaviour, and that the interaction is competitive. The obvious next questions are whether such behavioural shifts negatively affect co-occurring prey and whether cats will respond differently under a more sustained and intensive fox control regime. These questions are important to consider; cats have been incriminated in the extinction of many of the 29 species of mammals that have disappeared in Australia over the last 200 years, and of the > 100 extant native mammal species at risk of cat predation [16]; many occur in areas where fox control is implemented [40]. To reduce the likelihood of increased cat-impacts following fox control operations, it may therefore be necessary to implement targeted control of cats, ideally under an integrated pest management framework [2]. Indeed, our results suggest that cats should be targeted in open areas following fox control and/or in areas where cats have access to prey favoured by foxes (e.g. carcasses). Such management is likely to be especially important in regional areas from which the dingo has been removed and plays no role in suppressing the two mesopredators. Without effective control or removal of cats, fox control may not alleviate the impacts of predation on native fauna, but instead provide unexpected and potentially undesirable outcomes for conservation.

## References

[1] Newsome TM, Ripple WJ. A continental scale trophic cascade from wolves through coyotes to foxes. J Anim Ecol. 2015;84: 49–59. 10.1111/1365-2656.12258 24930631

[2] Glen AS, Dickman CR. Complex interactions among mammalian carnivores in Australia, and their implications for wildlife management. Biol Rev. 2005;80: 387–401. 16094805 10.1017/s1464793105006718

[3] Peterson RO. Wolves as interspecific competitors in canid ecology In: Carbyn LN, Fritts SH, Seip D, editors. Wolves in a Changing World. Edmonton (Canada): Canadian Circumpolar Institute, University of Alberta; 1996.

[4] Palomares F, Caro TM. Interspecific killing among mammalian carnivores. Am Nat. 1999;153: 492–508. 29578790 10.1086/303189

[5] Crooks KR, Soulé ME. Mesopredator release and avifaunal extinctions in a fragmented system. Nature. 1999;400: 563–566.

[6] Ritchie EG, Johnson CN. Predator interactions, mesopredator release and biodiversity conservation. Ecol Lett. 2009;12: 982–998. 10.1111/j.1461-0248.2009.01347.x 19614756

[7] Ripple WJ, Wirsing AJ, Wilmers CC, Letnic M. Widespread mesopredator effects after wolf extirpation. Biol Conserv. 2013;160: 70–79.

[8] Roemer GW, Gompper ME, Van Valkenburgh B. The ecological role of the mammalian mesocarnivore. BioScience. 2009;59: 165–173.

[9] Donadio E, Buskirk SW. Diet, morphology, and interspecific killing in carnivora. Am Nat. 2006;167: 524–536. 10.1086/501033 16670995

[10] Fancourt BA, Nicol SC, Hawkins CE, Cameron EZ, Jones ME. Devil declines and catastrophic cascades: is mesopredator release of feral cats inhibiting recovery of the eastern quoll. PloS One. 2015;10: e0119303 10.1371/journal.pone.0119303 25760348 PMC4356622

[11] Salo P, Banks PB, Dickman CR, Korpimäki E. Predator manipulation experiments: impacts on populations of terrestrial vertebrate prey. Ecol Monogr. 2010;80: 531–546.

[12] Crowther MS, Fillios M, Colman N, Letnic M. An updated description of the Australian dingo (Canis dingo Meyer, 1793). J Zool. 2014;293: 192–203.

[13] Schroeder T, Lewis MM, Kilpatrick AD, Moseby KE. Dingo interactions with exotic mesopredators: spatiotemporal dynamics in an Australian arid-zone study. Wildl Res. 2015;42: 529–539.

[14] Letnic M, Ritchie EG, Dickman CR. Top predators as biodiversity regulators: the dingo *Canis lupus dingo* as a case study. Biol Rev. 2012;87: 390–413. 10.1111/j.1469-185X.2011.00203.x 22051057

[15] Newsome TM, Ballard G-A, Crowther MS, Dellinger JA, Fleming PJS, Glen AS, et al Resolving the value of the dingo in ecological restoration. Restor Ecol. 2015;23: 201–208.

[16] Woinarski JCZ, Burbidge AA, Harrison PL. Ongoing unraveling of a continental fauna: Decline and extinction of Australian mammals since European settlement. Proc Natl Acad Sci. 2015;112: 4531–4540. 10.1073/pnas.1417301112 25675493 PMC4403217

[17] Catling PC. Similarities and contrasts in the diets of foxes, *Vulpes vulpes*, and cats, *Felis catus*, relative to fluctuating prey populations and drought. Aust Wildl Res. 1988;15: 307–317.

[18] Risbey DA, Calver MC, Short J. The impact of cats and foxes on the small vertebrate fauna of Heirisson Prong, Western Australia. I. Exploring potential impact using diet analysis. Wildl Res. 1999;26: 621–630.

[19] Glen AS, Pennay M, Dickman CR, Wintle BA, Firestone KB. Diets of sympatric native and introduced carnivores in the Barrington Tops, eastern Australia. Austral Ecol. 2011;36: 290–296.

[20] Christensen P, Burrows N. Project desert dreaming: the reintroduction of mammals to the Gibson Desert In: Serena M, editor. Reintroduction Biology of Australian and New Zealand Fauna. Chipping Norton: Surrey Beatty and Sons; 1995 pp. 199–208.

[21] Catling PC, Burt RJ. Why are red foxes absent from some eucalypt forests in eastern New South Wales? Wildl Res. 1995;22: 535–546.

[22] Read J, Bowen Z. Population dynamics, diet and aspects of the biology of feral cats and foxes in arid South Australia. Wildl Res. 2001;28: 195–203.

[23] Molsher RL. The ecology of feral cats, Felis catus, in open forest in New South Wales: interactions with food resources and foxes [Internet]. PhD Thesis, The University of Sydney. 1999. http://ses.library.usyd.edu.au/handle/2123/411

[24] Marlow NJ, Thomas ND, Williams AAE, Macmahon B, Lawson J, Hitchen Y, et al Cats (*Felis catus*) are more abundant and are the dominant predator of woylies (*Bettongia penicillata*) after sustained fox (*Vulpes vulpes*) control. Aust J Zool. 2015;63: 18–27.

[25] Davey C, Sinclair ARE, Pech RP, Arthur AD, Krebs CJ, Newsome AE, et al Do exotic vertebrates structure the biota of Australia? An experimental test in New South Wales. Ecosystems. 2006;9: 992–1008.

[26] Brunner H, Coman BJ. The Identification of Mammalian Hair. Melbourne: Inkata Press; 1974.

[27] Molsher RL, Newsome AE, Dickman CR. Feeding ecology and population dynamics of the feral cat (*Felis catus*) in relation to the availability of prey in central-eastern New South Wales. Wildl Res. 1999;26: 593–607.

[28] Molsher RL, Gifford EJ, McIlroy JC. Temporal, spatial and individual variation in the diet of red foxes (Vulpes vulpes) in central New South Wales. Wildl Res. 2000;27: 593–601.

[29] Nams VO. Locate II [Internet]. Pacer Computer Software. Tatamagouche, Nova Scotia, Canada; 1992. http://www.dal.ca/faculty/agriculture/environmental-sciences/faculty-staff/our-faculty/vilis-nams/locate-ii.html

[30] Kenward RE, Hodder KH. RANGES V: an analysis system for biological location data. Wareham, UK: Institute of Terrestrial Ecology; 1996.

[31] Molsher RL. Trapping and demographics of feral cats (Felis catus) in central New South Wales. Wildl Res. 2001;28: 631–636.

[32] Algar D, Smith R. Approaching eden. Landscope. 1998;13: 28–34.

[33] Thomas L, Buckland ST, Rexstad EA, Laake JL, Strindberg S, Hedley SL, et al Distance software: design and analysis of distance sampling surveys for estimating population size. J Appl Ecol. 2010;47: 5–14. 10.1111/j.1365-2664.2009.01737.x 20383262 PMC2847204

[34] Welsh AH, Lindenmayer DB, Donnelly CF. Fitting and interpreting occupancy models. PLoS ONE. 2013;8: e52015 10.1371/journal.pone.0052015 23326323 PMC3542396

[35] Macdonald DW. The ecology of carnivore social behaviour. Nature. 1983;301: 379–384.

[36] Newsome TM, Ballard G-A, Dickman CR, Fleming PJS, van de Ven R. Home range, activity and sociality of a top predator, the dingo: a test of the Resource Dispersion Hypothesis. Ecography. 2013;36: 914–925.

[37] Macdonald DW, Johnson DDP. Patchwork planet: the resource dispersion hypothesis, society, and the ecology of life. J Zool. 2015;295: 75–107.

[38] Durant SM. Competition refuges and coexistence: an example from Serengeti carnivores. J Anim Ecol. 1998;67: 370–386.

[39] Creel S. Four factors modifying the effect of competition on carnivore population dynamics as illustrated by African wild dogs. Conserv Biol. 2001;15: 271–274.

[40] Reddiex B, Forsyth DM, McDonald-Madden E, Einoder LD, Griffioen PA, Chick RR, et al Control of pest mammals for biodiversity protection in Australia. I. Patterns of control and monitoring. Wildl Res. 2006;33: 691–709.

